# Prescribing Trends of Antihypertensive Medications: An Observational Study in a Tertiary Care Hospital in India

**DOI:** 10.7759/cureus.101424

**Published:** 2026-01-13

**Authors:** Karan Suneja, Shalini Singh, Suyog Sindhu, Sarvesh Singh, Narendra Kumar, Amit Kumar, Satish Kumar, Ambuj Yadav, Deepak Bhagchandani, K K Sawlani

**Affiliations:** 1 Pharmacology and Therapeutics, King George's Medical University, Lucknow, IND; 2 Internal Medicine, King George's Medical University, Lucknow, IND

**Keywords:** acc/aha guidelines, antihypertensive drugs, hypertension, prescribing patterns, who prescribing indicators

## Abstract

Background and objectives: Hypertension is a major contributor to cardiovascular morbidity and mortality and represents a significant public health burden in India and blood pressure control rates remain low, contributing substantially to cardiovascular disease (CVD) risk. Appropriate pharmacological management at the time of diagnosis is essential for effective blood pressure control and prevention of complications. This study aimed to assess the demographic profile, comorbidities, and antihypertensive prescribing patterns among newly diagnosed hypertensive patients in a tertiary care hospital. The distribution of patients was classified according to the American College of Cardiology/American Heart Association (ACC/AHA) hypertension guidelines (2017), with consideration of subsequent updates, including the most recent 2025 recommendations, and prescribing trends based on World Health Organization (WHO) prescribing indicators were also analyzed.

Methods: This cross-sectional observational study included 250 adult patients newly diagnosed with hypertension. Data regarding demographic characteristics, lifestyle factors, body mass index (BMI), associated comorbidities, and prescribed antihypertensive medications were collected. Patients were categorized by age, BMI, and hypertension staging as per the latest ACC/AHA guidelines. Drug utilization patterns were evaluated using WHO prescribing indicators.

Results: The mean age of participants was 54.9 ± 13.0 years, and the mean BMI was 27.8 ± 5.9 kg/m². Individuals aged over 50 years constituted 60% of the study population, and approximately 70% were overweight or obese. Most patients were married, literate, and belonged to socio-economic class III, representing individuals with moderate educational attainment, semi-skilled or clerical occupations, and a middle-range household income relative to the urban population. Diabetes mellitus was the most common comorbidity (47.6%), followed by CVD (22.8%). Combination therapy was prescribed more frequently than monotherapy, with an average of 1.56 antihypertensive drugs per patient. Angiotensin receptor blockers (ARBs) were the most commonly prescribed agents. WHO indicators demonstrated rational prescribing, with high use of generic medicines and essential drugs.

Conclusion: The study highlights a high burden of hypertension among older, overweight, and diabetic individuals. Prescribing practices predominantly favored ARBs and reflected rational, guideline-consistent management of hypertension in a tertiary care setting.

## Introduction

Hypertension, commonly referred to as elevated blood pressure, is a major global public health concern and a significant contributor to the burden of non-communicable diseases (NCDs), particularly cardiovascular disease (CVD) [1,2]. Current estimates indicate that over one billion adults worldwide are affected by hypertension, a number projected to rise to 1.56 billion by 2025, with nearly 75% of the affected population residing in developing countries [3,4]. This growing burden is further compounded by poor medication adherence, as nearly half of patients discontinue antihypertensive therapy within the first year of treatment, significantly hindering effective blood pressure control [5]. Consequently, optimal blood pressure control is achieved in less than one-third of patients in high-income countries and only about one-tenth of patients in low- and middle-income countries [5]. In India, hypertension imposes a substantial burden on public health and healthcare systems due to its strong association with increased morbidity and mortality from stroke, ischemic heart disease, renal failure, congestive heart failure, and hypertensive heart disease [6]. India, a resource-limited country, reports a disproportionately high prevalence of hypertension, affecting nearly one in three adults, with an overall prevalence of 30.7% [7]. Similar findings, including marked urban-rural differences, have been reported in a systematic review by Anchala et al. [8]. The situation is further aggravated by low awareness levels, with a large proportion of individuals remaining unaware of their hypertensive status [9]. Hypertension often progresses silently and is frequently diagnosed incidentally or after the onset of target organ damage. Even among diagnosed individuals, limited access to healthcare and poor long-term treatment adherence remain significant challenges [1,2]. Evidence from a recent Chinese study demonstrated that nearly half of hypertensive patients were unaware of their condition, particularly younger individuals with fewer comorbidities, resulting in fewer opportunities for routine blood pressure screening [10]. Early detection and timely initiation of appropriate therapy are therefore critical for effective hypertension control and prevention of target organ damage. Several evidence-based clinical practice guidelines provide standardized recommendations for the diagnosis and management of hypertension. The American College of Cardiology/American Heart Association (ACC/AHA) hypertension guideline published in 2017 redefined blood pressure thresholds and emphasized risk-based management strategies [11]. Subsequent updates and reaffirmations through 2025 have reinforced these recommendations and reflect current evidence-based clinical practice [12]. The ACC/AHA guidelines recommend angiotensin-converting enzyme inhibitors, angiotensin receptor blockers (ARBs), calcium channel blockers, and thiazide or thiazide-like diuretics as first-line antihypertensive agents, either as monotherapy or in combination, based on blood pressure severity and overall cardiovascular risk [11,12]. Blood pressure targets are influenced by risk factors. However, deviations from guideline-based practice may occur due to factors such as clinician preference and limitations in healthcare resources, which may contribute to inappropriate prescribing patterns and therapeutic inertia. Moreover, converging evidence from large-scale clinical trials, pooled analyses, and contemporary guideline statements indicates that a substantial proportion of patients with hypertension require combination antihypertensive therapy to achieve recommended blood pressure targets, supporting the early consideration of multidrug, particularly single-pill, regimens to facilitate timely and sustained blood pressure control [13]. The benefits of intensive blood pressure lowering have also been shown to vary according to comorbidity burden, with greater benefits observed in patients with mild to moderate comorbidities such as uncomplicated diabetes mellitus, early-stage chronic kidney disease, or stable CVD, whereas high comorbidity burden may refer to individuals with multiple, advanced, or poorly controlled chronic conditions, including advanced chronic kidney disease, heart failure, or significant frailty [14]. Despite the availability of multiple treatment options and well-established guidelines, real-world data on antihypertensive prescribing patterns in India, particularly from North India, remain limited. Understanding these patterns is essential for evaluating prescribing appropriateness, identifying deviations from evidence-based recommendations, and improving therapeutic outcomes. Accordingly, the present study was undertaken to assess real-world prescribing trends of antihypertensive medications among patients attending a tertiary care hospital in India.

## Materials and methods

Study design and setting

This was a cross-sectional observational study conducted over a period of one year at King George’s Medical University, Lucknow, Uttar Pradesh, India. Data were collected from the Medicine Outpatient Department of the Department of General Medicine, in collaboration with the Department of Pharmacology and Therapeutics. The diagnosis of hypertension was established by the treating physician based on office-based blood pressure measurements obtained during routine outpatient care, in line with prevailing clinical practice. 

Inclusion and exclusion criteria

Adult patients aged ≥18 years of either gender who were newly diagnosed with hypertension and prescribed at least one oral antihypertensive medication were included in the study after obtaining informed consent. Patients were excluded if they did not provide informed consent or had pregnancy-induced hypertension, hypertensive emergencies, or malignant hypertension. Individuals with known secondary hypertension attributable to renal or hepatic disease were excluded. Additionally, patients with a history of substance abuse, those with cognitive impairment, or individuals who were dependent on others for medication administration were excluded from the study.

Data collection

The study population consisted of newly identified adult patients with hypertension attending the Medicine outpatient department during the study period with hypertension who met the eligibility criteria and attended the Medicine Outpatient Department of the General Medicine at King George’s Medical University, Lucknow, were invited to participate in the study. Written informed consent was obtained from all participants after explaining the objectives of the study. Confidentiality and anonymity of patient information were strictly maintained throughout the data collection process. Relevant demographic and clinical data, along with details of prescribed antihypertensive medications, were recorded at the time of patient evaluation

Sample size

The sample size was calculated using the standard formula for determining sample size in prevalence studies.

The formula used was as follows:

$n = \frac{Z^{2} P (1 - P)}{d^{2}}$

where n represents the required sample size, Z is the Z statistic corresponding to the desired confidence level, P is the expected prevalence, and d is the precision. Based on a study [15] conducted by Roy et al. in 2019 in Odisha, India, the reported incidence of adverse drug reactions (ADRs) to antihypertensive medications was 30.71%. Accordingly, P was taken as 0.3071, with a 95% confidence level (Z = 1.96) and a relative precision of 20%. Using these parameters, the calculated minimum sample size was 216.7, which was rounded up to 217. After accounting for a 15% dropout rate, the final sample size was increased to 250 patients. Therefore, a minimum of 250 patients was included in the study.

Outcome variables

The primary outcome variables included prescribing trends of antihypertensive medications, frequency of monotherapy and combination therapy, and utilization patterns of different pharmacological classes of antihypertensive drugs. Compliance with World Health Organization (WHO) prescribing indicators [16] and standard hypertension management guidelines [17] was also assessed.

Statistical analysis

The collected data were compiled and analyzed using IBM SPSS Statistics software, version 24.0 (IBM Corp., Armonk, NY, USA). The analyses were performed using an institutionally licensed free version of the software. Descriptive statistics were used for data analysis. Continuous variables such as age and BMI were expressed as mean ± standard deviation (SD), while categorical variables such as gender, grades of hypertension, and prescribed antihypertensive drug classes were presented as frequencies and percentages. Data were presented using tables. 

## Results

Demographic, clinical, and sociodemographic profile

The study comprised newly identified adult patients with hypertension, defined as individuals without a prior diagnosis of hypertension who were identified for the first time during the study period based on elevated office-based blood pressure measurements obtained during routine evaluation in the Medicine outpatient department. The mean age of the participants was 55 years, indicating a predominance of middle-aged and elderly individuals in the study population. As depicted in Table 1, the highest proportion of patients belonged to the age group above 50 years (60%), demonstrating an age-related increase in the prevalence of hypertension in the study population. The mean BMI of the participants was 27.81 ± 5.95 kg/m², reflecting that a majority were either overweight or obese. The distribution of BMI categories according to the WHO [18] classification is shown in Table 2, presenting the distribution of patients according to body mass index categories. Sociodemographic characteristics are summarized in Table 3. Most participants were married (97.6%), literate (98%), and belonged to the middle socio-economic class (96.8%), which categorizes socioeconomic status based on education, occupation, and monthly family income. Socio-economic status classes II, III, and IV according to the Modified Kuppuswamy scale, which is based on a composite score derived from education, occupation, and monthly family income. Specifically, Class II (upper-middle) represents higher educational attainment and skilled or professional occupation with higher income; Class III (lower-middle) represents moderate educational attainment, semi-skilled occupation, and intermediate income; and Class IV (upper-lower) represents lower educational attainment, unskilled or semi-skilled occupation, and lower income. Lifestyle-related risk factors such as tobacco use and alcohol consumption were infrequently reported in the study population.

**Table 1:** Age-wise distribution of the study population

Age Group (years)	Frequency (n)	Percentage (%)
18–30	20	8%
31–50	80	32%
>50	150	60%

**Table 2:** BMI of the patients in the study Source: [18]

BMI Category	Frequency (n)	Percentage (%)
Underweight (<18.5)	5	2%
Normal weight (18.5–24.9)	70	28%
Overweight (25–29.9)	100	40%
Obese (≥30)	75	30%

**Table 3:** Socio-demographic details of the study group

Variable	Category	Frequency (n)	Percentage (%)
Marital status	Married	244	97.6
	Unmarried	6	2.4
Literacy	Literate	245	98%
	Illiterate	5	2%
Socio-economic status (Class)	2	3	1.2
	3	242	96.8
	4	5	2%

Comorbid conditions

A substantial proportion of patients had associated comorbidities. As outlined in Table 4, diabetes mellitus was the most frequently observed comorbidity (47.6%), followed by CVDs (22.8%). Other conditions included hypothyroidism, chronic obstructive pulmonary disease, dyslipidemia, and miscellaneous disorders, indicating a high burden of concurrent metabolic and cardiovascular illnesses among hypertensive individuals

**Table 4:** Comorbidities of the study population

S.No	Comorbidities	No (N)	Yes	Percentage (%)
1	Diabetes mellitus	131	119	47.6%
2	Hypothyroidism	238	12	4.8%
3	Osteoarthritis	248	2	0.8%
4	Peptic ulcer disease	250	0	0%
5	Dyslipidemia	246	4	1.6%
6	Chronic obstructive pulmonary disease	243	7	2.8%
7	Cardiovascular disease	193	57	22.8%
8	Other diseases	200	50	20%

Antihypertensive prescribing pattern and blood pressure status

All enrolled patients received antihypertensive therapy. The distribution of prescribed antihypertensive drug classes is presented in Table 5. ARBs were the most frequently prescribed agents (63.6%), followed by calcium channel blockers (34.8%) and diuretics (30%). Combination therapy was prescribed more often (56.4%) than monotherapy (43.6%), with an average of 1.56 antihypertensive drugs per patient. The duration of therapy varied, with the majority of patients receiving treatment for four weeks, as shown in Table 6. Blood pressure categories were defined according to the ACC/AHA 2017 hypertension guidelines, with updates through 2025, based on recorded systolic and diastolic blood pressure values [17] as summarized in Table 7, indicating that a considerable proportion of patients presented were having raised blood pressure or were in stage 1 or stage 2 hypertension, thereby necessitating combination pharmacotherapy.

**Table 5:** Prescribing pattern of drugs

S.No	Drug Class	Patients Prescribed (n)	Percentage (%)
1	Angiotensin receptor blocker	159	63.60%
2	Angiotensin-converting enzyme inhibitor	9	3.60%
3	Calcium channel blocker	87	34.80%
4	Diuretic	75	30%
5	Beta-blocker	39	15.60%
6	Other agents	51	20.40%
7	Monotherapy	109	43.60%
8	Combination therapy	141	56.40%
	Average number of drugs per patient	-	1.56

**Table 6:** Time interval of prescribing antihypertensive medications

Duration of Therapy	Frequency (n)	Percentage (%)
1 week	2	0.8%
2 weeks	19	7.6%
3 weeks	5	2%
4 weeks	183	73.2%
8 weeks	41	16.4%

**Table 7:** Grading of hypertension ACC: American College of Cardiology; AHA: American Heart Association; BP: blood pressure; SBP: systolic blood pressure; DBP: diastolic blood pressure Source: [17]

ACC/AHA BP Category	BP Threshold (mmHg)	Frequency (n)	Percentage (%)
Elevated BP	SBP 120–129 and DBP <80	107	42.8
Stage 1 hypertension	SBP 130–139 or DBP 80–89	74	29.6
Stage 2 hypertension	SBP ≥140 or DBP ≥90	69	27.6

WHO prescribing indicators

Assessment of prescribing practices using WHO prescribing indicators [16] is shown in Table 8. All encounters involved the prescription of at least one antihypertensive drug. A large proportion of medications were prescribed by their generic names (85%), and the majority were selected from the essential medicines list (92%). No injectable antihypertensive agents were prescribed. 

**Table 8:** WHO Prescribing guidelines %: percentage Source: [16]

S.No	WHO Prescribing Indicators	Value/Observation
1	Average number of antihypertensive drugs per patient	1.56
2	% of encounters with at least one drug prescribed	100%
3	% of patients on monotherapy	43.6%
4	% of patients on combination therapy	56.4%
5	% prescribed angiotensin receptor blocker	63.6%
6	% prescribed angiotensin-converting enzyme inhibitor	3.6%
7	% prescribed calcium channel blocker	34.8%
8	% prescribed diuretics	30%
9	% prescribed beta-blocker	15.6%
10	% prescribed other antihypertensive agents	20.4%
11	% drugs prescribed by generic name	85%
12	% encounters with injections	0%
13	% drugs from the essential medicines list	92%

Prescription analysis using WHO prescribing indicators

Prescription practices were evaluated in accordance with the WHO prescribing indicators [16] to determine the rationality of drug use and compliance with standard treatment guidelines. As summarized in Table 8, all prescriptions contained at least one antihypertensive agent, reflecting adherence to appropriate therapeutic protocols. The average number of drugs per prescription suggested rational polytherapy, consistent with the treatment requirements of the study population. In the present study, evaluation using WHO prescribing indicators showed a high proportion of drugs prescribed by generic name and selected from the Essential Medicines List, reflecting adherence to principles of rational prescribing. In addition, the absence of injectable antihypertensive formulations suggests a preference for oral therapy, consistent with WHO recommendations for the management of chronic conditions. Importantly, the predominant use of first-line antihypertensive drug classes aligns with the ACC/AHA 2017 hypertension guidelines and subsequent updates through 2025, which recommend angiotensin-converting enzyme inhibitors, ARBs, calcium channel blockers, and thiazide or thiazide-like diuretics as initial therapy. While these findings suggest concordance with contemporary guideline-based practice, interpretation should be considered in the context of the cross-sectional design and the use of categorical prescribing indicators. Prescribing practices in the present study demonstrated substantial use of generic medicines and Essential Medicines List drugs, with no injectable antihypertensive agents prescribed, in line with WHO prescribing indicators and national guidelines.

## Discussion

The present study provides an analytical overview of prescribing patterns among newly identified hypertensive patients, with a focus on drug utilization assessed using WHO prescribing indicators. While these indicators primarily evaluate prescribing behavior rather than clinical outcomes, the findings suggest that prescribers predominantly utilized antihypertensive drug classes recommended as first-line therapy in contemporary guidelines. However, the study does not assess blood pressure control, hypertension severity, or cardiovascular risk stratification, which limits interpretation regarding the appropriateness of treatment intensity. Current ACC/AHA hypertension guidelines (2017 with updates through 2025) emphasize a risk-based approach to treatment initiation, including consideration of patients with systolic blood pressure values between 130 and 139 mmHg based on overall cardiovascular risk.

A predominance of middle-aged and elderly patients was observed in the study population, which may reflect the age distribution of individuals presenting to outpatient services during the study period rather than a direct association with hypertension onset. Similar age-related trends have been consistently reported in large epidemiological studies, attributing this association to progressive vascular stiffness and endothelial dysfunction with aging [19,20]. The higher prevalence of hypertension among individuals aged over 50 years observed in this study aligns with contemporary global and regional data, which demonstrate a sharp rise in blood pressure levels beyond midlife [19,21]. A relatively high proportion of participants in the present study were classified as overweight or obese. While excess body weight is a well-recognized modifiable factor associated with hypertension through mechanisms such as neurohumoral activation, insulin resistance, and altered renal sodium handling, the cross-sectional design of the study limits causal inference.

In addition, socio-demographic variables, including socio-economic status and educational level, were assessed; however, their potential role as confounding factors could not be fully evaluated within the scope of the present analysis. Comparative assessment with regional or population-based data would be required to determine whether the study sample is representative of the general population. Furthermore, the use of a tertiary care outpatient setting may have introduced selection bias, as such centers often manage patients with different demographic and clinical profiles compared with the broader community [22,23]. These demographic characteristics are consistent with national and international trends reported in similar clinical settings [21,24]. Comorbid conditions, particularly diabetes mellitus and cardiovascular disease, were frequently observed in the present study, highlighting the close interrelationship between metabolic and cardiovascular disorders. Recent evidence emphasizes that hypertension commonly clusters with diabetes due to shared pathophysiological pathways, including insulin resistance, chronic inflammation, and endothelial dysfunction [25,26]. This coexistence underscores the need for comprehensive management strategies that address both blood pressure control and metabolic regulation. In contrast, the relatively lower prevalence of other comorbidities, such as hypothyroidism, chronic obstructive pulmonary disease, and dyslipidemia, suggests that hypertension in this population predominantly coexists with metabolic disorders, a finding consistent with contemporary observational studies [26,27]. Although first-line antihypertensive agents were commonly prescribed, the relatively low average number of medications per patient and the absence of blood pressure outcome data preclude conclusions regarding treatment adequacy, particularly in the context of contemporary evidence supporting more intensive therapy for optimal blood pressure control. ARBs were the most commonly prescribed antihypertensive agents, followed by calcium channel blockers and diuretics.

This prescribing pattern is in agreement with recent international hypertension guidelines, including those issued by the International Society of Hypertension and the European Society of Cardiology, which recommend these drug classes as first-line therapy for newly diagnosed hypertension [28,29]. The frequent use of ARBs in the present study may reflect their established efficacy and favorable tolerability profile, as well as their perceived benefits in patients with diabetes or cardiovascular comorbidities [29]. The use of calcium channel blockers and diuretics, either as monotherapy or in combination, is consistent with their recommended role as first-line antihypertensive agents in contemporary guidelines, rather than indicating achievement of optimal blood pressure control [28]. Evaluation using WHO prescribing indicators further supports the rationality of the observed prescribing practices. The average number of antihypertensive drugs per prescription was relatively low in the present study. While this may limit concerns related to polypharmacy, the absence of blood pressure control data precludes assessment of whether treatment intensity was sufficient to achieve guideline-recommended targets. Contemporary evidence suggests that many patients require combination therapy to attain adequate blood pressure control, and in this context, single-pill combination regimens play an important role by enabling treatment intensification while minimizing pill burden. Most medications were prescribed by their generic names and selected from the Essential Medicines List, reflecting cost-effective prescribing behavior. The absence of injectable formulations is appropriate for outpatient hypertension management and aligns with WHO recommendations for rational drug use. Collectively, these findings indicate a high level of awareness among prescribers regarding evidence-based, safe, and economical pharmacotherapy.

The study has several strengths. Inclusion of newly diagnosed hypertensive patients minimized confounding related to prior antihypertensive exposure. The systematic assessment using WHO prescribing indicators enabled an objective evaluation of rational drug use, while the integration of demographic and pharmacological data provided a comprehensive overview of real-world hypertension management practices. However, certain limitations must be acknowledged. As a single-center, cross-sectional study conducted in a tertiary care setting, the findings may not be generalizable to primary healthcare facilities or community-based populations. The absence of follow-up data limits the evaluation of long-term blood pressure control, treatment adherence, and adverse drug reactions. Additionally, prescription audits do not capture the clinical reasoning underlying individualized therapeutic decisions. Despite these limitations, the methodological rigor, structured analysis, and alignment with WHO prescribing indicators enhance the credibility of the study findings. In a broader context, this study adds to the literature describing contemporary antihypertensive prescribing patterns in clinical practice. The rational selection of first-line agents, preference for generic and essential medicines, and avoidance of unnecessary medications reflect progress toward effective and economically sustainable hypertension management [16]. These findings underscore the importance of regular prescription audits, continuous medical education, and dissemination of updated clinical guidelines as integral components of hypertension control programs, ultimately improving quality of care and optimizing healthcare resource utilization.

## Conclusions

In this study population, a predominance of middle-aged and older adults was observed among individuals identified with hypertension, and a higher proportion of participants were classified as overweight or obese. The frequent coexistence of diabetes mellitus and CVD underscores the need for integrated management strategies that address both blood pressure control and associated metabolic conditions. The prescribing patterns observed were rational and aligned with established clinical guidelines, with ARBs, calcium channel blockers, and diuretics constituting the principal classes of antihypertensive medications used. Furthermore, the high proportion of prescriptions containing essential and generic medicines, along with the absence of irrational polypharmacy, reflects strong adherence to World Health Organization standards for rational prescribing. Although the study is limited by its cross-sectional design and single-center setting, it provides valuable insights into current prescribing practices and highlights encouraging progress toward evidence-based hypertension management. Ongoing physician education, reinforcement of treatment guidelines, and regular prescription audits may further strengthen rational drug use and contribute to improved quality of care and long-term cardiovascular outcomes.
